# Association between Perfluoroalkyl Substances in Follicular Fluid and Polycystic Ovary Syndrome in Infertile Women

**DOI:** 10.3390/toxics12020104

**Published:** 2024-01-26

**Authors:** Sen Li, Guojing Li, Yu Lin, Feng Sun, Liqiang Zheng, Yingying Yu, Hong Xu

**Affiliations:** 1 International Peace Maternity & Child Health Hospital, School of Medicine, Shanghai Jiao Tong University, Shanghai 200030, China; lisen194@foxmail.com (S.L.); liguojing0907@163.com (G.L.); linyu89@mail.ustc.edu.cn (Y.L.); sunfeng0711@126.com (F.S.); 2 Shanghai Municipal Key Clinical Speciality, Shanghai 200030, China; 3 School of Public Health, School of Medicine, Shanghai Jiao Tong University, Shanghai 200025, China; zhenglq@sjtu.edu.cn

**Keywords:** perfluoroalkyl substances, perfluorooctanoic acid, polycystic ovary syndrome, risk factor

## Abstract

In recent years, perfluoroalkyl substances (PFASs), a family of fluorinated organic com pounds, have garnered much attention due to their reproductive and developmental toxicity in humans. Polycystic ovary syndrome (PCOS) is a prevalent endocrine disease that affects women of reproductive age and is a significant contributor to female infertility. A previous study suggested that PFASs play a possible role in PCOS. We conducted a clinical study investigating the relationship between PCOS and PFAS in follicular fluid. A total of 73 infertile patients with PCOS and 218 controls were recruited from the International Peace Maternity and Child Health Hospital, affiliated with the Shanghai Jiao Tong University School of Medicine. The concentrations of 12 PFASs in follicular fluid samples and sex hormones in serum were measured. Correlation analysis and multiple linear regression revealed a positive relationship between perfluorooctanoic acid (PFOA) and testosterone (T) concentrations. The adjusted odds ratios (ORs) and 95% confidence intervals (CIs) for each PFAS were estimated using multivariable logistic regression and quantile-based g-computation (QGC). The PFOA concentrations in follicular fluid were correlated with increased odds of PCOS (second vs. first quartile: OR = 3.65, 95% CI: 1.47–9.05, *p* = 0.005; third vs. first quartile: OR = 2.91, 95% CI: 1.17–7.26, *p* = 0.022; fourth vs. first quartile: OR = 3.13, 95% CI: 1.21–8.09, *p* = 0.019; P for trend = 0.032). This association was confirmed with QGC. Mediation analysis suggested that the mediation effect of T in association with PFOA and PCOS was not statistically significant. Our study suggests that PFOA may be a risk factor for PCOS.

## 1. Introduction

Per- and polyfluoroalkyl substances (PFASs) are a family of environmental toxicants that are ubiquitously present and pose a constant exposure risk to humans; examples include perfluorocarboxylic acids (PFCAs) and perfluoroalkyl sulfonic acids (PFSAs) [1]. Due to their distinctive characteristics, such as thermal stability, hydrophobicity, and oleophobicity, PFASs have been extensively employed in various industries [1,2]. These applications encompass a wide range of industries, such as the manufacturing of nonstick cookware, textiles with oil and water resistance, food packaging that is resistant to grease, surfactants, fire-fighting foams, pesticides, cosmetics, and various construction and electronic products [2]. PFASs exhibit exceptional stability and resistance to degradation, enabling their accumulation in the food chain [2]. These compounds, particularly perfluorooctane sulfonate (PFOS) and PFOA, have been consistently identified in wildlife and human specimens on a global scale. Human exposure to these compounds arises from both direct and indirect sources, encompassing contaminated food and drinking water, subsequently leading to dermal contact and inhalation [3]. The global detection of PFASs at a high prevalence in humans has been documented, leading to concerns regarding the potential adverse health effects linked to exposure to these substances. In recent years, there has been a growing body of experimental and epidemiological research indicating that PFASs may function as endocrine-disrupting chemicals (EDCs) [4]. These substances can disrupt endocrine processes and pose potential risks to reproductive and developmental health [4].

Polycystic ovary syndrome (PCOS) is a prevalent endocrine disorder that affects a significant number of women in their reproductive years [5]. This abnormality, known for its manifestations of elevated androgen levels, disturbances in ovulation, and the presence of polycystic ovaries, is a prominent factor contributing to infertility, affecting approximately 80% of women experiencing anovulatory infertility [6,7]. The etiology of this disease remains incompletely elucidated; however, multiple lines of evidence have suggested that PCOS arises from a complex interplay between genetic predisposition and environmental factors [7,8]. PFASs, due to their ability to disrupt the endocrine system, have the potential to contribute to the pathogenesis of PCOS. Nevertheless, the existing body of evidence regarding the relationship between PCOS and PFAS exposure is limited and inconclusive.

In 2009, the Stockholm Convention on Persistent Organic Pollutants (POPs) classified PFASs as compounds with “restricted use” [9]. However, a significant portion of the production of these chemicals continues to be carried out in China [10]. China stands out as one of the few remaining manufacturers and the primary user of PFASs, making it the most prominent location for contamination [11,12]. This study aimed to investigate the potential connections between PFAS levels in follicular fluid and PCOS in China. By doing so, we aimed to enhance our comprehension of the reproductive health implications of PFAS exposure in women.

## 2. Materials and Methods

### 2.1. Study Population

To assess the association of PFAS exposure with PCOS, this case-control study was carried out on infertile women between 2020 and 2021. Infertility was defined as the inability to conceive naturally despite engaging in regular and unprotected sexual intercourse for a duration exceeding 12 months. After excluding other possible causes of infertility, 73 women diagnosed with PCOS were considered as the case group. PCOS was defined according to the Rotterdam criteria, encompassing the presence of ovulatory dysfunction, the characteristic appearance of polycystic ovarian morphology, and biochemical and clinical hyperandrogenism, while excluding other specific diseases [13]. Three normal women who underwent assisted reproductive technology due to male reproductive dysfunction during the same period were recruited for each PCOS patient as controls (*n* = 219). A control woman was excluded because of an insufficient follicular fluid sample. All participants had no chromosomal abnormalities. Before the study, all participants provided their consent after being informed. The study protocol was approved by the Ethics Committees of the International Peace Maternity and Child Health Hospital affiliated with the Shanghai Jiao Tong University School of Medicine (Approval Number: GKLW 2017-77), and all procedures were in line with the principles stated in the Declaration of Helsinki.

### 2.2. Data Collection

Participants were guided by a trained assistant to complete a standardized survey to collect personal details, including demographic characteristics (age, height, weight, employment status, education level, etc.), lifestyle behaviors (history of drinking and smoking), companions’ lifestyle behaviors (history of smoking), and reproductive history (history of pregnancy and delivery). Medical examination information, including gynecological examinations and chromosome analysis, was collected from medical records.

### 2.3. Measurement of PFASs in Follicular Fluid and Sex Hormones in Serum

Follicular fluid samples were collected on the day of the women’s ovum pick-up (OPU). After oocyte extraction, clear follicular fluid without discernible blood contamination was deposited on ice. The initial aliquot of clear follicular fluid was discarded due to the possibility of contamination with the wash media used in the OPU-tubing system. All follicular fluid samples from a single patient were combined and centrifuged at 500× *g*. Before analysis, the supernatant was kept at −80 °C.

Using an ultra-performance liquid chromatography (UPLC)–tandem Q Exactive Orbitrap mass spectrometer (Thermo Fisher Scientific, Waltham, MA, USA), the following twelve PFASs were measured in follicular fluid: perfluorooctanoic acid (PFOA), perfluorodecanoic acid (PFDA), perfluorononanoic acid (PFNA), perfluoroundecanoic acid (PFUnDA), perfluoroheptanoic acid (PFHpA), perfluorododecanoic acid (PFDoDA), perfluorooctane sulfonate (PFOS), perfluorobutanesulfonic acid (PFBS), perfluorodecane sulfonic acid (PFDS), perfluorohexane sulfonate (PFHxS), perfluoroheptanesulfonic acid (PFHpS), and perfluorooctanoic sulfonamide (PFOSA). Detailed PFAS measurement procedures have been reported elsewhere [14]. The limit of detection (LOD) for the chosen PFASs was determined at a signal-to-noise ratio of 10 and ranged from 0.10 ng/mL to 10.00 ng/mL.

On days 1–3 of the menstrual cycle, baseline hormone profiles of follicle-stimulating hormone (FSH), luteinizing hormone (LH), estradiol (E2), progesterone (P), testosterone (T), and prolactin (PRL) were regularly measured in the venous blood serum.

### 2.4. Statistical Analysis

Continuous variables are expressed as the mean, standard deviation (SD) or median, interquartile range (IQR). A *t*-test was used to compare the differences in variables with normal distributions between PCOS cases and controls, and Wilcoxon’s rank sum test was employed for variables with skewed distributions. A chi-square test was used to analyze the differences in the categorical variables. PFAS concentrations below the LOD were set to a value equivalent to the LOD divided by the square root of 2. Spearman’s correlation and multivariate linear regression were employed to assess the correlation between PFAS concentrations and sex hormone levels. To account for multiple comparisons, adjustments were made using the Benjamini and Hochberg false discovery rate (FDR) method (FDR < 0.05). Furthermore, extreme gradient boosting (XGBoost) was employed to examine potential interactions among the PFASs. The H statistic was utilized to depict possible chemical interactions, with a scale ranging from 0 to 1 [15]. A higher value of the H statistic suggests a stronger interaction between the features. PFAS concentrations were categorized into four quartiles (first, second, third, and fourth quartiles). Unconditional logistic regression was applied to assess the relevance of PCOS and PFAS levels to estimate the odds ratio (OR) and 95% confidence interval (95% CI) for each PFAS quartile, with the first quartile as the reference. The potential confounding factors in multivariate logistic analysis were selected according to previous research and univariate logistic analysis results. The relationships between the potential confounding factors are described using directed acyclic graphs (DAGs) (Supplementary Figure S1). In premenopausal women, PFASs are eliminated through menstrual bleeding [16]. Zhou et al. showed that certain PFASs were associated with menstrual abnormalities [17]. In this study, menstrual volumes were difficult to quantify, and the length of menstrual cycles varied widely; therefore, these two covariates were potential confounders. Because none of the participants drank alcohol, drinking was also not included in our present study. Because the relationship between BMI and PCOS is complex, we also performed a logistic regression analysis without having BMI as a covariate.

Bayesian kernel machine regression (BKMR), a semi-parametric approach for evaluating the effects of complex environmental pollutants, was employed to assess potential nonlinearity and interactions among exposures [18,19]. The exposure–response connection was fitted for binary outcomes using a probit link function. To adjust for skewness and reduce the impact of extreme values and varied value scales in the variables, PFAS concentrations were transformed using a natural logarithm centered to a mean of 0 and scaled to a standard deviation of 1 for all BKMR analyses. The Gaussian kernel was selected to express the dose–response function. Posterior inclusion probabilities (PIPs) were the values that reflected relative variable importance calculated with the Markov chain Monte Carlo (MCMC) algorithm, and 50,000 iterations were selected. To demonstrate the interactions between PFAS mixtures and the particular surfaces of dose–response functions, we performed the following analysis: (1) all of the other PFAS concentrations were fixed at the 50th percentile, and the univariate relationship of each PFAS with PCOS was explored; (2) estimates of all predictors at a specific percentile (ranging from 0.25 to 0.75) were compared with their 50th percentile estimates to derive the mixed total effect; (3) the remaining PFAS concentrations were fixed at 0.25, 0.50, and 0.75, and the contribution of each PFAS to PCOS was calculated; and (4) changes in the effects of individual PFAS exposures when all of the other PFAS concentrations were fixed at the 25th and 75th percentiles were explored.

Quantile-based g-computation (QGC) is a recently established arithmetic method that builds weight indices within the overall framework of marginal structural models to ascertain the cumulative effects of various chemicals [20]. This arithmetic method combines the flexibility of g-computation with the inferential simplicity of weighted quantile regression (WQS) and the nonlinearity and nonadditivity of BKMR, which seems less biased and more reliable. In this study, QGC was employed to assess the overall joint association of PFASs.

Mediation analysis was used to quantify the required magnitude of the mediation effect. In our study, direct effect indicated an association between PFAS and PCOS; indirect effect indicated an association between PFAS and PCOS that was mediated by T. The proportion mediated represented the percentage of the mediating effect.

The statistical analyses were conducted using SPSS version 26 and R software (version 4.2.1, R Development Core Team, 2022). The mediation analysis, BKMR model, QGC, and XGBoost were implemented using the R packages “mediation”, “bkmr”, “qgcomp”, and “xgboost”, respectively [19,21]. *p* < 0.05 was considered statistically significant.

## 3. Results

### 3.1. Characteristics of the Study Population in Terms of Demographics

The main demographic characteristics of the 73 PCOS cases and 218 controls are shown in Table 1. The age and BMI status distributions were significantly distinct between the PCOS-related cases and controls (*p* < 0.05). Compared with the group with PCOS, the control group showed a higher percentage of pregnancy and delivery. The control group had a lower rate of active/passive smoking (*p* = 0.003). There were no discernible distinctions between the two groups regarding employment status or educational level. Women with PCOS had much higher LH and T levels than controls (*p* < 0.001) but significantly lower FSH levels (*p* < 0.05).

### 3.2. The Concentrations of PFASs in Follicular Fluid

The concentrations and detection rates of PFASs in the follicular fluid of the study participants are shown in Table 2. PFOSA, PFHpA, PFDS, and PFBS were discovered in fewer than 50% of the participants and thus were removed from the following analysis. Eight of the twelve PFASs evaluated in the 291 samples were detected at concentrations above the LOD (Table 2).

PFOA had the highest concentration, followed by PFOS. The PCOS cases had significantly lower median levels of PFDoDA than the controls (*p* = 0.019) (Table 2). The concentrations of the other PFASs in the follicular fluid were comparable across the two study groups.

Most PFASs in follicular fluid were significantly correlated with each other (*p* < 0.05). The coefficients ranged from 0.146 to 0.913, according to Spearman’s correlation analysis (Table 3). It is worth noting that the longer-chain PFCAs, such as PFNA, PFDA, and PFUnDA, exhibited stronger correlation with the other PFASs (coefficients: 0.847 to 0.913). Based on the results of XGBoost, there are identifiable interactions among the PFASs (Figure S2).

### 3.3. Association between PFASs in Follicular Fluid and Hormone Levels in Serum

Tables S1 and S2 present the results of the correlation analysis. After the FDR adjustment, no significant correlation was observed between the concentrations of PFASs in follicular fluid and sex hormones in the controls. After adjusting for confounding variables in multivariate linear regression among the controls, PFOA and PFNA levels in follicular fluid were significantly positively correlated with T concentration in serum (*p* < 0.05) (Table 4).

### 3.4. The Relationship between PFAS Levels in Follicular Fluid and PCOS

Table 5 summarizes the connections between PFAS levels and PCOS. In the crude analysis, no significant relationship was observed between the level of each PFAS and PCOS (*p* > 0.05).

By controlling for age, BMI, employment status, educational level, active/passive smoking, pregnancy, and delivery, multivariable-adjusted logistic regression analyses were conducted to assess the associations between PFAS concentrations in follicular fluid and PCOS. As indicated in Table 5, the PFOA concentration in follicular fluid was significantly correlated with an elevated risk of PCOS (second vs. first quartile: OR = 3.65, 95% CI: 1.47–9.05, *p* = 0.005; third vs. first quartile: OR = 2.91, 95% CI: 1.17–7.26, *p* = 0.022; fourth vs. first quartile: OR = 3.13, 95% CI: 1.21–8.09, *p* = 0.019; P for trend = 0.032). The adjusted model found no statistically significant association between PCOS and other PFAS concentrations.

In the analysis without controlling for BMI, the dose−effect relationship between PFOA levels and the risk of PCOS was more significant in the model (second vs. first quartile: OR = 3.98, 95% CI: 1.64–9.69, *p* = 0.002; third vs. first quartile: OR = 3.39, 95% CI: 1.38–8.30, *p* = 0.008; fourth vs. first quartile: OR = 3.20, 95% CI: 1.27–8.12, *p* = 0.014; P for trend = 0.016) (Supplementary Table S3).

### 3.5. BKMR-Based Models of the Association of Individual and Joint PFAS Exposure with PCOS

**Figure 1:**
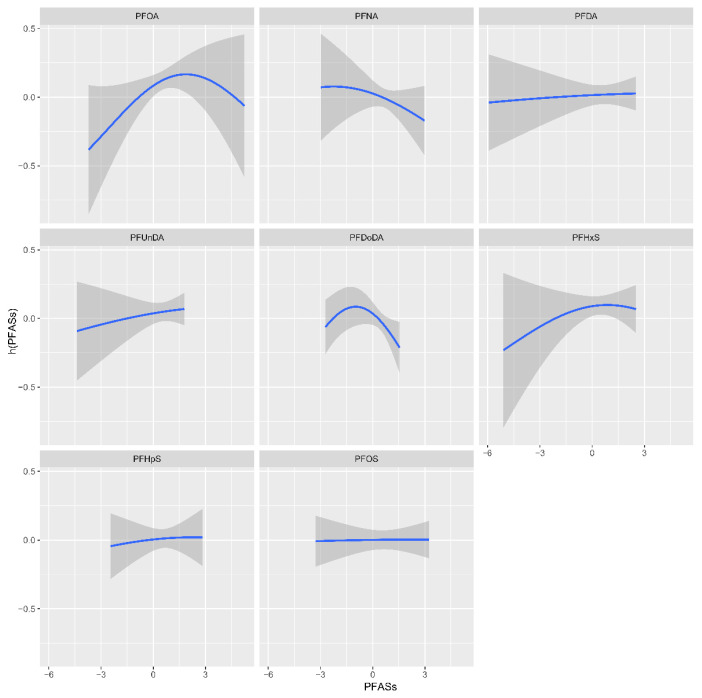
The univariate concentration–response functions for each PFAS with 95% confidence bands (shaded regions), with the other PFASs maintained at the median.

By setting the other PFAS concentrations to the 50th percentile, we evaluated the univariate connection between each PFAS and PCOS. The results are shown in Figure 1.

Figure S3 shows the results of the overall relation between the PFAS mixture (estimates and 95% confidence intervals) and PCOS in the BKMR models. There was no significant association between the risk of PCOS and increasing concentrations of total PFAS mixture (Supplementary Figure S3).

### 3.6. Mixture Analyses Using the Quantile-Based g-Computation Method

The results of the quantile-based g-computation analyses for the risk of PCOS are shown in Table 6. The PFOA concentration was significantly positively associated with PCOS in all participants with an OR of 1.74 (95% CI: 1.17–2.64, *p* = 0.007). Furthermore, the PFOA concentration also showed a positive correlation with PCOS, with an OR of 3.46 (95% CI: 1.20–13.56, *p* = 0.044), while PFDoDA showed a negative correlation, with an OR of 0.25 (95% CI: 0.08 = 0.58, *p* = 0.004) in overweight and obese women (BMI ≥ 24 kg/m^2^).

### 3.7. The Mediation Effect of Testosterone in the Relationship between PFOA and PCOS

**Figure 2:**
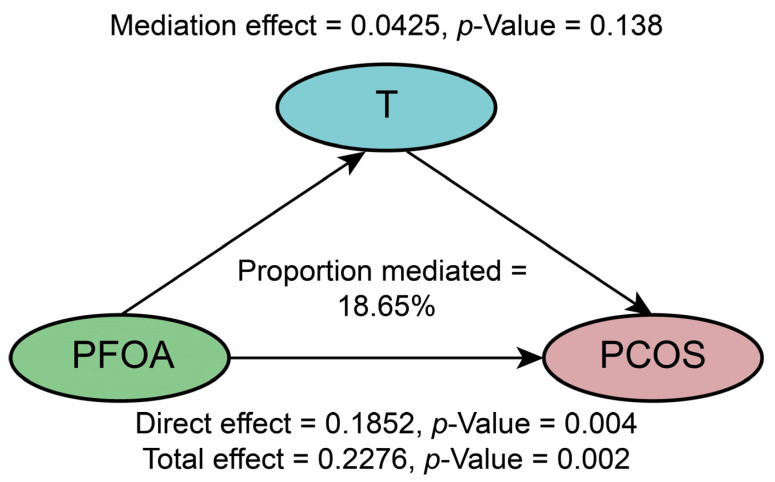
Path diagram of the mediation analysis of T in the relationship between PFOA and PCOS.

We further analyzed the mediating role of T in the association of PCOS and PFOA exposure. The results are shown in Figure 2. T explained 18.65% of the association between PFOA and PCOS. The direct effect of PFOA was significant (*p* = 0.004); however, the mediation effect of T was not statistically significant (*p* = 0.138) (Figure 2).

## 4. Discussion

In this case-control study, we explored the relationship between PFAS levels in follicular fluid and PCOS. After adjusting for potential confounders, PFOA concentration in follicular fluid was significantly associated with elevated odds of PCOS.

Most previous studies have chosen serum or urine samples because they are easier to obtain. Kang et al. demonstrated that the concentration of PFASs in serum and follicular fluid does not differ significantly, and several PFASs exhibit a high transfer efficiency between serum and follicular fluid [22]. However, follicular fluid is the microenvironment for the survival of endocrine cells and oocytes in the ovary. Compared with urine and blood samples, the impact of PFAS in follicular fluid on the female reproductive endocrine system is direct and significant. However, the possible reproductive health impacts of PFOA exposure and the underlying mechanisms warrant additional exploration in the future. The potential impact of PFASs, particularly PFOA and PFOS, on the environment and human health has resulted in a slew of regulations and restrictions on their production and use in a variety of organizations and countries, bolstering a global shift toward the adoption of less-harmful alternatives [23,24].

In the present study, we measured the concentrations of PFASs in the follicular fluid of 73 women with PCOS and 218 controls. The median PFOA concentration in the follicular fluid of the PCOS patients was higher than that in the controls, although the observed discrepancy was not statistically significant. This result was consistent with a previous study by Vagi et al. and contrasts with studies by Wang et al. and Heffernan et al. [25,26,27]. These discrepant results between different studies are probably attributable to the differences in the menstrual characteristics the participants. Because long-chain PFASs have lengthy half-lives and strong binding affinities to plasma proteins, menstruation may play a significant role in regulating PFAS concentrations in premenopausal women [28,29]. A number of previous studies measured the concentrations of PFASs in umbilical cord blood and maternal blood from pregnant women and demonstrated that select PFASs could pass the placental barrier and reach the developing fetus [30,31,32,33,34,35,36,37]. In women of reproductive age, the expulsion of the placenta, fetus, and other tissues during parturition is believed to be an important way to remove PFASs from the body [38]. In the present study, the proportion of parous women was much higher in the controls than the cases. This may also have led to differences in the PFAS concentrations in follicular fluid between the cases and controls. A Norwegian pregnancy cohort study supports this hypothesis [39]. According to Vagi et al., PCOS risk was considerably enhanced by higher concentrations of PFOA in serum [25]. In our study, we also observed similar findings in follicular fluid.

An increasing number of studies have demonstrated the biological toxicity of PFOA. Animal studies have shown that PFOA and PFOS can affect the spleen, bone marrow, thymus, and other immune organs, as well as nonspecific immune functions such as the protective skin barrier, protective intestinal mucosal barrier, and humoral immunity [40]. PFOA exposure might affect the reproductive function of male rodents [41,42,43]. Exposure to PFOA can also reduce ovarian reserve function in women [44]. PFOA might also be related to the occurrence of many diseases, such as ulcerative colitis, kidney cancer, and testicular cancer [45,46,47]. At the recent International Agency for Research on Cancer (IARC) in Lyon, scientists proposed that PFOA and its corresponding isomers and salts may be involved in the occurrence of human cancer by inducing epigenetic alterations and suppressing immune function [48]. Furthermore, PFOA and PFOS can attack the thyroid gland, leading to hypothyroidism, which is more common in women and children [49]. PFASs in the urine or serum are also associated with various endocrine disorders, such as PCOS and endometriosis [25,26]. Several studies have also observed an association between serum PFASs and their alternatives and free T in serum in adolescents and women of reproductive age [50,51]. This phenomenon was also observed in our study. In order to ascertain the potential mediation effect of T in the association between PFOA and PCOS, we conducted a mediation analysis. The results suggest that PFOA is more likely to be involved in the pathogenesis of PCOS directly rather than through the mediation of T. In the present study, the results of QGC showed that PFDoDA was negatively correlated with PCOS, while this phenomenon was not observed in women with a BMI < 24 kg/m^2^. In a previous report by Wang et al., there was a positive association between the PFDoDA concentration in serum and PCOS in selected individuals [26]. Different samples (serum vs. follicular fluid) might explain the different results. PFAS in serum can be transferred to follicular fluid, and the concentration of PFAS in serum is always positively correlated with the concentration in follicular fluid [22]. Furthermore, Wang et al. did not conduct a subgroup analysis on overweight women, which might be the reason for the opposite results. The disparities in the outcomes between the study by Wang et al. and ours may also likely be caused by women’s different menstrual features and parity histories. The participants in Wang’s research were nulliparous women, and some of our participants had already delivered a baby. Wang et al. also adjusted for menstrual characteristics as confounding factors. Additional studies are needed to confirm the potential impact of PFDoDA exposure on women with PCOS.

In a previous clinical study in Zhejiang province conducted with infertile women, PFBS concentrations in plasma were found to be correlated with an increased risk of infertility related to endometriosis, whereas the PFOA concentration in plasma did not show a significant relationship. These disparities might be partly attributed to the research population’s varying levels and compositions of PFASs, which might contribute to the underlying processes in the etiology of PCOS- or endometriosis-related infertility.

Significant evidence indicates that exposure to PFASs might affect female reproductive health. The potential mechanisms may include interference with the hypothalamic–pituitary–gonadal axis or the metabolic composition of follicular fluid [44,52,53,54,55,56]. However, the hypothesized reproductive toxicities of PFASs in humans are still being debated due to inconsistencies in human epidemiological evidence [57]. As previously stated, we discovered several differences between the current study and other comparable papers. It is worth noting that the present study found a positive relationship between PFOA exposure and PCOS. Further investigation into the possible effects of this chemical on the metabolic composition of follicular fluid is needed.

In our study, a pronounced correlation was observed among various PFAS concentrations, particularly among PFCAs (Table 3). Similar results have been reported in other studies as well [26,58]. This suggests that people might be exposed to PFASs in a similar exposure pattern. The gradual accumulation of PFASs in different organs may also contribute to the observed correlations [59]. Furthermore, the similar structures and chemical properties among different PFASs might result in comparable metabolic pathways within the biological system, contributing to the observed correlation [60]. It is noteworthy that strong correlations among long-chain PFCAs, i.e., PFNA, PFDA and PFUnDA, was observed, whereas PFOA was less well correlated with them. Similar observations were reported in the study conducted by Harada et al. [61]. The authors speculated that there might be special exposure sources of long-chain PFCAs. Further research is needed to elucidate the potential mechanisms. The correlation between different PFASs makes it difficult to hypothesize a specific causality between the levels of a specific PFAS and a specific condition or disease. Conversely, this could be beneficial for investigating approaches to effectively eliminate multiple types of PFASs from the human body simultaneously.

Our study measured PFAS concentrations in follicular fluid. The effect of PFAS in follicular fluid on female reproductive function is direct, and the analysis results were more significant. Nevertheless, we did not measure the serum PFAS concentrations and conduct a correlation analysis between PFAS concentrations in blood and follicular fluid, which is a limitation of our study. Furthermore, obtaining qualified follicular fluid samples was difficult, which limited our ability to include more patients in our study. We also collected detailed information on pregnancy and delivery for all participants. However, residual confounding by unmeasured variables is still possible, which could have biased our results. For example, menstrual characteristics, the use of oral contraceptives (OCs), and the characteristics of OC use may alter the concentrations of PFASs in follicular fluid. In addition, daily habits such as drinking water and the use of industrial products can also affect the intake and accumulation of PFASs in the human body. Prospective population studies taking into account more possible confounders are required to investigate the involvement of PFASs in the etiology of female infertility and PCOS.

## 5. Conclusions

In our present study, the median concentration of PFDoDA in PCOS cases was significantly lower than that in healthy controls. Moreover, according to the multivariable-adjusted logistic regression analysis and QGC, PFOA in follicular fluid correlated with a heightened risk of PCOS. PFOA might play a direct role in the occurrence of PCOS. These discoveries have increased our understanding of the harmful effects of PFOA exposure and raised concerns about the impact of exposure to long-carbon-chain PFCAs on reproductive health. Further research is needed to understand these effects fully.

## Figures and Tables

**Table 1:** Demographic characteristics of the study population (*n* = 291).

	Controls (*n* = 218)	Cases (*n* = 73)	*p*-Value
	*n* (%) or Mean (SD)	*n* (%) or Mean (SD)	
Age (years)	35.4 (5.0)	32.4 (4.0)	0.000
BMI (kg/m^2^)			0.002
<18.5	18 (8.3)	5 (6.8)	
18.5–23.9	146 (67.0)	35 (47.9)	
24–27.9	44 (20.2)	21 (28.8)	
≥28	10 (4.6)	12 (16.4)	
Employment status			0.236
Unemployed	18 (8.3)	3 (4.1)	
Employed	200 (91.7)	70 (95.9)	
Educational level			0.839
≤High school	80 (36.7)	24 (32.9)	
College graduate	116 (53.2)	41 (56.2)	
>College graduate	22 (10.1)	8 (11.0)	
Active/passive smoking			0.003
No	142 (70.0)	61 (83.6)	
Yes	76 (34.9)	12 (16.4)	
Pregnancy			0.000
0	97 (44.5)	57 (78.1)	
≥1	121 (55.5)	16 (21.9)	
Delivery			0.002
0	175 (80.3)	70 (95.9)	
≥1	43 (19.7)	3 (4.1)	
FSH	8.3 (3.4)	7.2 (1.7)	0.030
LH	4.1 (1.9)	9.5 (5.5)	0.000
E2	134.2 (64.4)	140.5 (79.4)	0.840
P	1.9 (2.4)	1.7 (0.9)	0.372
T	1.3 (0.7)	2.3 (1.1)	0.000
PRL	16.0 (12.0)	17.3 (19.3)	0.657

**Table 2:** Follicular fluid concentrations of PFASs in the study population (*n* = 291).

PFASs	Measure ≥ LOD *	Median (IQR)			
ng/mL	(%)	Controls (*n* = 218)	Cases (*n* = 73)	Total (*n* = 291)	*p*-Value
PFOA	100	6.90 (4.64, 9.96)	7.22 (5.84, 9.74)	7.04 (5.05, 9.84)	0.316
PFNA	100	1.04 (0.68, 1.55)	1.00 (0.74, 1.34)	1.04 (0.70, 1.49)	0.475
PFDA	99.6	1.18 (0.76, 2.02)	1.20 (0.65, 1.80)	1.19 (0.70, 1.92)	0.586
PFUnDA	99.0	0.70 (0.39, 1.16)	0.65 (0.38, 0.97)	0.67 (0.39, 1.09)	0.509
**PFDoDA**	**89.7**	**0.17 (0.12, 0.23)**	**0.15 (0.08, 0.19)**	**0.16 (0.11, 0.22)**	**0.019**
PFHxS	99.6	1.41 (0.74, 2.71)	1.44 (0.97, 2.86)	1.41 (0.87, 2.84)	0.661
PFHpS	92.8	0.09 (0.05, 0.14)	0.09 (0.05, 0.12)	0.09 (0.05, 0.14)	0.323
PFOS	100	6.04 (4.06, 9.27)	5.48 (3.69, 7.80)	5.99 (3.94, 9.11)	0.265
∑PFAS		(12.44, 27.68)	17.74 (13.61, 24.00)	18.67 (13.20, 26.45)	

* LOD (ng/mL): PFOA (0.01), PFNA (0.01), PFDA (0.01), PFUnDA (0.01), PFDoDA (0.01), PFHxS (0.01), PFHpS (0.01), PFOS (0.1).

**Table 3:** Spearman’s correlation coefficients between follicular fluid concentrations of PFASs.

	PFOA	PFNA	PFDA	PFUnDA	PFDoDA	PFHxS	PFHpS
**PFNA**	0.677 **						
**PFDA**	0.523 **	0.886 **					
**PFUnDA**	0.471 **	0.847 **	0.913 **				
**PFDoDA**	0.323 **	0.575 **	0.591 **	0.594 **			
**PFHxS**	0.563 **	0.460 **	0.400 **	0.275 **	0.146 *		
**PFHpS**	0.621 **	0.744 **	0.684 **	0.596 **	0.426 **	0.553 **	
**PFOS**	0.564 **	0.829 **	0.882 **	0.790 **	0.540 **	0.431 **	0.823 **

* *p* < 0.05; ** *p* < 0.001.

**Table 4:** Associations between PFASs and hormones in multiple linear regression in the controls.

	FSH	LH	E2	P	T	PRL
PFOA **	−0.08 (−0.22, 0.05)	−0.06 (−0.20, 0.07)	0.10 (−0.03, 0.24)	−0.02 (−0.16, 0.12)	0.18 (0.05, 0.31) *	0.03 (−0.11, 0.17)
PFNA **	−0.10 (−0.23, 0.03)	−0.001 (−0.14, 0.13)	−0.02 (−0.15, 0.12)	0.001 (−0.14, 0.14)	0.16 (0.03, 0.28) *	0.14 (0.00, 0.27)
PFDA **	−0.12 (−0.25, 0.01)	−0.05 (−0.18, 0.08)	−0.007 (−0.14, 0.12)	−0.01 (−0.15, 0.13)	0.11 (−0.02, 0.23)	0.08 (−0.05, 0.22)
PFUnDA **	−0.09 (−0.22, 0.04)	0.009 (−0.12, 0.14)	−0.05 (−0.19, 0.08)	−0.03 (−0.16, 0.11)	0.10 (−0.03, 0.23)	0.11 (−0.02, 0.24)
PFDoDA **	−0.08 (−0.21, 0.05)	−0.09 (−0.23, 0.04)	−0.007 (−0.14, 0.13)	−0.04 (−0.18, 0.10)	0.03 (−0.10, 0.16)	−0.02 (−0.15, 0.12)
PFHxS **	−0.01 (−0.14, 0.12)	−0.02 (−0.16, 0.11)	−0.04 (−0.18, 0.09)	0.13 (−0.008, 0.26)	0.05 (−0.07, 0.18)	0.02 (−0.11, 0.16)
PFHpS **	−0.07 (−0.20, 0.06)	0.03 (−0.10, 0.16)	0.02 (−0.11, 0.15)	0.08 (−0.05, 0.22)	0.07 (−0.06, 0.19)	0.06 (−0.07, 0.20)
PFOS **	−0.08 (−0.21, 0.04)	0.000 (−0.13, 0.13)	−0.03 (−0.16, 0.10)	0.05 (−0.08, 0.19)	0.08 (−0.05, 0.21)	0.03 (−0.10, 0.16)

* *p* < 0.05; ** Adjusted for age, BMI, employment status, educational level, active/passive smoking, pregnancy, and delivery.

**Table 5:** Odds ratios for follicular fluid concentrations of PFASs associated with PCOS (*n* = 291).

PFASs	Quartiles	Crude OR	*p*-Value	Adjusted OR *	*p*-Value
	(ng/mL)	(95% CI)		(95% CI)	
**PFOA**	1st (≤5.05)	1.00 (reference)		**1.00 (reference)**	
	2nd (>5.05, 7.04)	3.09 (1.35, 7.05)	0.008	**3.65 (1.47, 9.05)**	**0.005**
	3rd (>7.04, 9.84)	2.59 (1.12, 6.00)	0.026	**2.91 (1.17, 7.26)**	**0.022**
	4th (>9.84)	2.06 (0.88, 4.84)	0.097	**3.13 (1.21, 8.09)**	**0.019**
	P for trend **	0.053		**0.032**	
**PFNA**	1st (≤0.70)	1.00 (reference)		1.00 (reference)	
	2nd (>0.70, 1.04)	1.64 (0.78, 3.44)	0.192	2.55 (1.10, 5.90)	0.029
	3rd (>1.04, 1.49)	1.28 (0.60, 2.74)	0.530	1.50 (0.63, 3.59)	0.364
	4th (>1.49)	0.92 (0.42, 2.04)	0.840	1.18 (0.49, 2.83)	0.718
	P for trend **	0.419		0.134	
**PFDA**	1st (≤0.70)	1.00 (reference)		1.00 (reference)	
	2nd (>0.70, 1.19)	0.49 (0.23, 1.08)	0.076	0.56 (0.23, 1.38)	0.210
	3rd (>1.19, 1.92)	1.11 (0.55, 2.25)	0.770	1.38 (0.63, 3.01)	0.423
	4th (>1.92)	0.60 (0.28, 1.28)	0.185	0.68 (0.30, 1.56)	0.364
	P for trend **	0.115		0.194	
**PFUnDA**	1st (≤0.39)	1.00 (reference)		1.00 (reference)	
	2nd (>0.39, 0.67)	1.27 (0.62, 2.63)	0.512	1.51 (0.66, 3.44)	0.329
	3rd (>0.67, 1.09)	1.11 (0.54, 2.32)	0.774	1.18 (0.52, 2.71)	0.694
	4th (>1.09)	0.57 (0.25, 1.28)	0.173	0.70 (0.28, 1.72)	0.435
	P for trend **	0.237		0.367	
**PFDoDA**	1st (≤0.11)	1.00 (reference)		1.00 (reference)	
	2nd (>0.11, 0.16)	0.86 (0.42, 1.73)	0.669	0.88 (0.39, 1.95)	0.747
	3rd (>0.16, 0.22)	0.63 (0.30, 1.32)	0.220	0.62 (0.27, 1.43)	0.265
	4th (>0.22)	0.42 (0.19, 0.92)	0.030	0.46 (0.19, 1.11)	0.084
	P for trend **	0.145		0.309	
**PFHxS**	1st (≤0.87)	1.00 (reference)		1.00 (reference)	
	2nd (>0.87, 1.41)	1.82 (0.84, 3.92)	0.127	2.38 (0.97, 5.79)	0.057
	3rd (>1.41, 2.84)	1.40 (0.64, 3.10)	0.399	2.17 (0.87, 5.41)	0.095
	4th (>2.84)	1.48 (0.68, 3.24)	0.324	2.04 (0.85, 4.88)	0.11
	P for trend **	0.502		0.226	
**PFHpS**	1st (≤0.05)	1.00 (reference)		1.00 (reference)	
	2nd (>0.05, 0.09)	1.06 (0.51, 2.19)	0.880	1.38 (0.61, 3.10)	0.436
	3rd (>0.09, 0.14)	1.00 (0.48, 2.09)	0.990	1.13 (0.49, 2.61)	0.782
	4th (>0.14)	0.65 (0.30, 1.42)	0.281	0.95 (0.40, 2.26)	0.905
	P for trend **	0.611		0.823	
**PFOS**	1st (≤3.94)	1.00 (reference)		1.00 (reference)	
	2nd (>3.94, 5.99)	0.73 (0.35, 1.52)	0.401	1.20 (0.52, 2.78)	0.671
	3rd (>5.99, 9.11)	0.86 (0.42, 1.78)	0.692	0.98 (0.43, 2.22)	0.953
	4th (>9.11)	0.55 (0.26, 1.18)	0.127	0.73 (0.31, 1.71)	0.469
	P for trend **	0.471		0.732	

* Adjusted for age, BMI, employment status, educational level, active/passive smoking, pregnancy, and delivery; ** *p*-value for a test of a trend across quartiles.

**Table 6:** The overall effect of the exposure mixture and separate effect of individual PFAS after correcting for other PFAS congeners on PCOS, stratified by weight status.

PCOS			
Items	Weight	[OR (95% CI)]	*p*-Value
All subjects * **(*n* = 291)**			
PFAS mixture		0.96 (0.73, 1.26)	0.771
**PFOA**	**0.56**	**1.74 (1.17, 2.64)**	**0.007**
PFNA	−0.08	0.92 (0.48, 1.75)	0.802
PFDA	0.42	1.52 (0.74, 3.22)	0.260
PFUnDA	−0.38	0.68 (0.37, 1.24)	0.209
PFDoDA	−0.32	0.73 (0.51, 1.03)	0.075
PFHxS	0.05	1.05 (0.74, 1.49)	0.795
PFHpS	−0.10	0.90 (0.55, 1.46)	0.674
PFOS	−0.20	0.82 (0.46, 1.45)	0.489
BMI < 24 kg/m^2^ ** **(*n* = 204)**			
PFAS mixture		1.20 (0.80, 1.80)	0.369
PFOA	0.47	1.59 (0.97, 2.65)	0.067
PFNA	−0.49	0.61 (0.26, 1.37)	0.245
PFDA	0.63	1.87 (0.75, 4.90)	0.187
PFUnDA	−0.34	0.71 (0.33, 1.48)	0.367
PFDoDA	0.003	1.00 (0.66, 1.54)	0.988
PFHxS	0.09	1.10 (0.70, 1.70)	0.682
PFHpS	0.08	1.08 (0.55, 2.14)	0.821
PFOS	−0.18	0.84 (0.39, 1.77)	0.642
BMI ≥ 24 kg/m^2^ ** **(*n* = 87)**			
PFAS mixture		0.79 (0.49, 1.28)	0.339
**PFOA**	**1.24**	**3.46 (1.20, 13.56)**	**0.044**
PFNA	−0.44	0.64 (0.09, 4.36)	0.650
PFDA	−0.50	0.61 (0.08, 4.10)	0.605
PFUnDA	0.76	2.14 (0.49, 10.23)	0.315
**PFDoDA**	**−1.40**	**0.25 (0.08, 0.58)**	**0.004**
PFHxS	−0.08	0.93 (0.45, 1.93)	0.836
PFHpS	−0.58	0.56 (0.19, 1.50)	0.253
PFOS	0.42	1.53 (0.40, 5.94)	0.531

* QGC model adjusting for age, BMI, employment status, educational level, active/passive smoking, pregnancy, and delivery; ** QGC model adjusting for age, BMI, employment status, educational level, active/passive smoking, pregnancy, and delivery.

## Data Availability

The data presented in this study are available on request from the corresponding author. The data are not publicly available due to another study based on the data has not been completed yet.

## Supplementary Materials

The following supporting information can be downloaded at: https://www.mdpi.com/article/10.3390/toxics12020104/s1. Figure S1: Directed acyclic graphs (DAGs). Figure S2: Interaction of individual PFASs in the associations with PCOS in XGBoost analysis. Estimates were adjusted for women’s age, BMI, employment status, educational level, active/passive smoking, pregnancy, and delivery. Figure S3: The cumulative impact of PFASs (estimates with 95% confidence intervals). PFASs are at a particular percentile (x-axis) compared with when all exposures are at the 50th percentile. Table S1: Spearman’s correlation coefficients between follicular fluid concentration of PFASs and hormones in serum in the control group (*n* = 218). Table S2: *p* values for Spearman’s correlation coefficients between follicular fluid concentration of PFASs and hormones in serum in the control group after Benjamini and Hochberg (BH) false discovery rate (FDR) adjustment. Table S3: Odds ratios for follicular fluid concentrations of PFASs associated with PCOS without adjustment for BMI (*n* = 291).
