# Supplementary material for: Association between Perfluoroalkyl Substances in Follicular Fluid and Polycystic Ovary Syndrome in Infertile Women
Source: Toxics. 2024 Jan 26;12(2):104. doi: 10.3390/toxics12020104 (PMC10893032; doi:10.3390/toxics12020104)
Supplement: Supplementary file 1 [file toxics-12-00104-s001.zip › toxics-2812077-supplementary.pdf]

# Association between perfluoroalkyl substances in follicular fluid and polycystic ovary syndrome in infertile women

Sen Li [1, 2, †], Guojing Li [1, 2, †], Yu Lin [1, 2], Feng Sun [1, 2], Liqiang Zheng [3], Yingying Yu [1, 2, *], Hong Xu [1, 2, *]

[†] These two authors contributed equally to this work and should be considered co-first authors.

[1] International Peace Maternity & Child Health Hospital, School of Medicine, Shanghai Jiao Tong University, Shanghai, China.

[2] Shanghai Municipal Key Clinical Speciality, Shanghai, China.

[3] School of Public Health, School of Medicine, Shanghai Jiao Tong University, Shanghai, China.

[*] Corresponding author:

Hong Xu, MD, PhD, Address: No.910 Hengshan Road, Xuhui District, Shanghai, China. Telephone: +86-18017316266.

E-mail: xuhong1558@sjtu.edu.cn.

Yingying Yu, MD, PhD, Address: No.910 Hengshan Road, Xuhui District, Shanghai, China. Telephone: +86-13564702091

E-mail: yu-ying-ying@163.com.

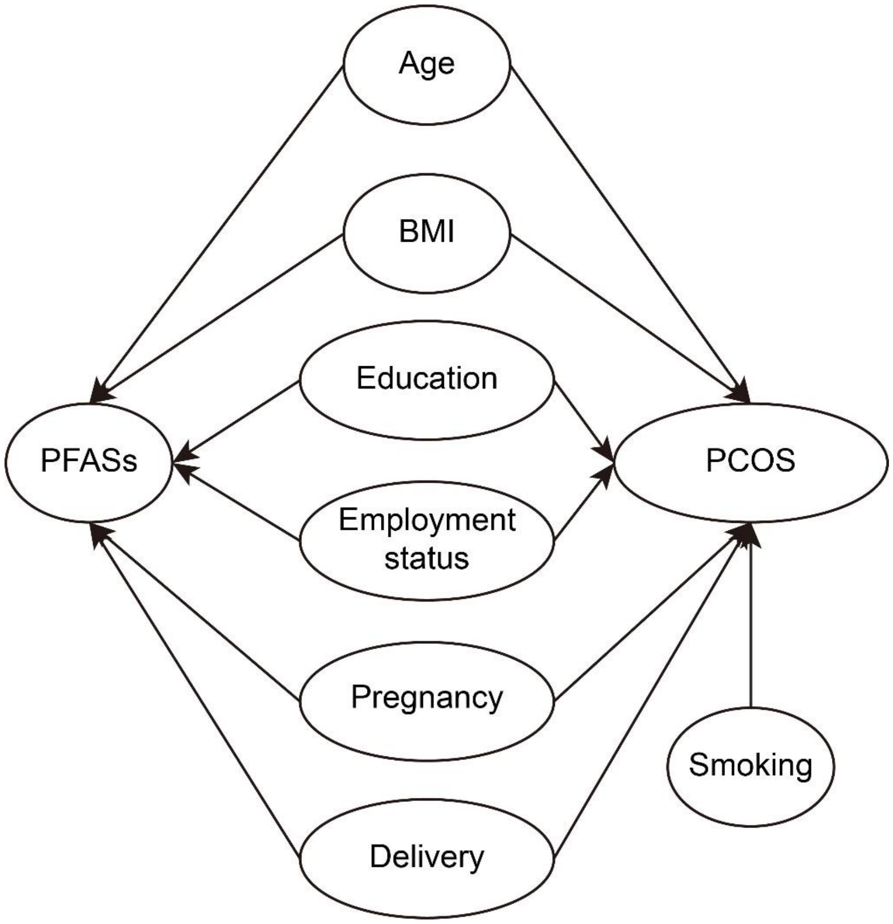


**Fig. S1.** Directed acyclic graphs (DAGs)

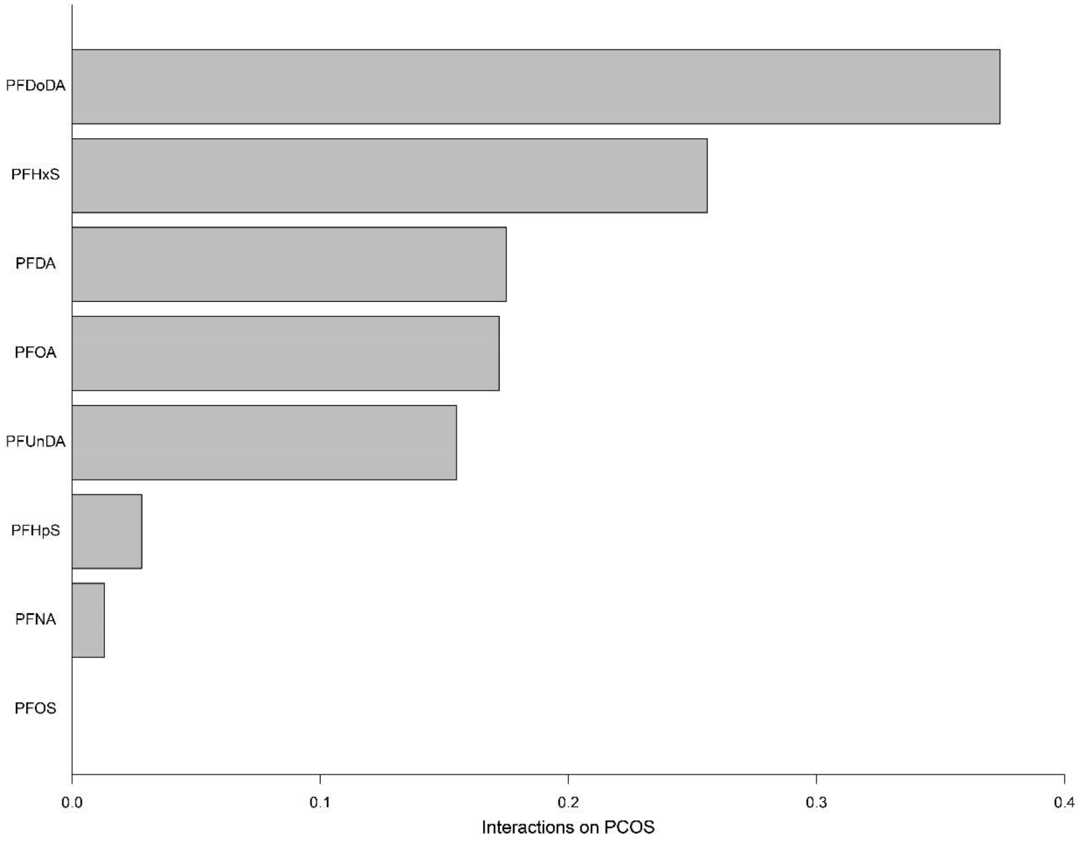


**Fig. S2.** Interaction of individual PFAS in the associations with PCOS in XGBoost analysis. Estimates were adjusted for women's age, BMI, employment status, educational level, active/passive smoking, pregnancy and delivery.

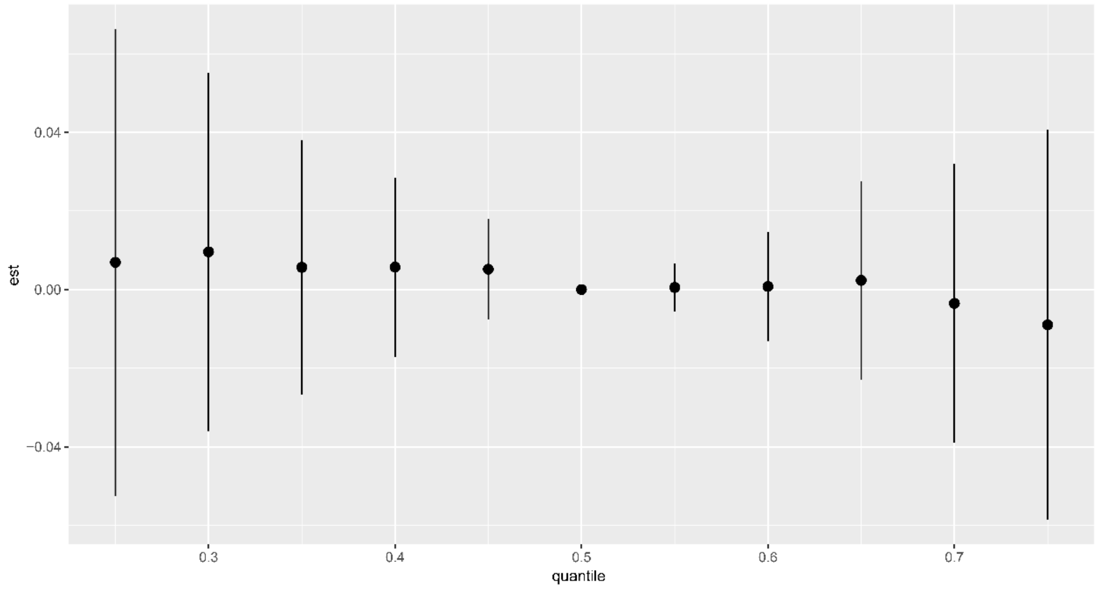


**Fig. S3.** The cumulative impact of PFASs (estimates with 95% confidence intervals). PFASs are at a particular percentile (X-axis) compared to when all exposures are at the 50th percentile.

**Table S1** Spearman correlation coefficients between follicular fluid concentration of PFASs and hormone in serum in the control group (n = 218).

| | FSH | LH | E2 | P | T | PRL |
|---|---|---|---|---|---|---|
| PFOA | -0.094 | 0.056 | 0.022 | 0.031 | 0.124 | 0.131 |
| PFNA | -0.050 | 0.070 | 0.006 | 0.072 | 0.127 | 0.131 |
| PFDA | -0.022 | 0.047 | 0.008 | 0.019 | 0.113 | 0.138* |
| PFUnDA | -0.023 | 0.128 | 0.039 | 0.008 | 0.091 | 0.126 |
| PFDoDA | -0.002 | -0.066 | 0.043 | -0.086 | 0.002 | 0.006 |
| PFHxS | -0.013 | 0.038 | -0.026 | 0.083 | 0.065 | 0.130 |
| PFHpS | -0.030 | 0.098 | 0.051 | 0.113 | 0.103 | 0.135* |
| PFOS | 0.003 | 0.043 | 0.022 | 0.069 | 0.101 | 0.161* |

Abbreviations: FSH, follicle stimulating hormone, LH, luteinizing hormone, E2, estradiol, P, progesterone, T, testosterone, PRL, prolactin.* $P<0.05$

**Table S2** P values for spearman correlation coefficients between follicular fluid concentration of PFASs and hormone in serum in the control group after Benjamini and Hochberg (BH) false discovery rate (FDR) adjustment.

| | FSH | LH | E2 | P | T | PRL |
|---|---|---|---|---|---|---|
| PFOA | 0.50 | 0.79 | 0.92 | 0.92 | 0.32 | 0.32 |
| PFNA | 0.83 | 0.68 | 0.98 | 0.68 | 0.32 | 0.32 |
| PFDA | 0.92 | 0.84 | 0.98 | 0.93 | 0.38 | 0.32 |
| PFUnDA | 0.92 | 0.32 | 0.86 | 0.98 | 0.50 | 0.32 |
| PFDoDA | 0.98 | 0.68 | 0.85 | 0.55 | 0.98 | 0.98 |
| PFHxS | 0.98 | 0.86 | 0.92 | 0.56 | 0.68 | 0.32 |
| PFHpS | 0.92 | 0.48 | 0.83 | 0.38 | 0.47 | 0.32 |
| PFOS | 0.98 | 0.92 | 0.92 | 0.68 | 0.47 | 0.32 |

**Table S3** Odds ratios for follicular fluid concentrations of PFASs associated with PCOS s without adjustment for BMI (n = 291).

| PFASs | Quartiles (ng/mL) | Adjusted OR (95% CI) | P-value |
|---|---|---|---|
| PFOA | 1st (≤5.05) | 1.00 (reference) | |
| | 2nd ( > 5.05, 7.04) | 3.98 (1.64, 9.69) | 0.002 |
| | 3rd ( > 7.04, 9.84) | 3.39 (1.38, 8.30) | 0.008 |
| | 4th ( > 9.84) | 3.20 (1.27, 8.12) | 0.014 |
| | P for trend** | 0.016 | |
| PFNA | 1st (≤0.70) | 1.00 (reference) | |
| | 2nd ( > 0.70, 1.04) | 2.48 (1.10, 5.62) | 0.029 |
| | 3rd ( > 1.04, 1.49) | 1.75 (0.76, 4.02) | 0.186 |
| | 4th ( > 1.49) | 1.12 (0.48, 2.63) | 0.798 |
| | P for trend** | 0.108 | |
| PFDA | 1st (≤0.70) | 1.00 (reference) | |
| | 2nd ( > 0.70, 1.19) | 0.68 (0.30, 1.57) | 0.368 |
| | 3rd ( > 1.19, 1.92) | 1.41 (0.66, 3.03) | 0.374 |
| | 4th ( > 1.92) | 0.68 (0.31, 1.52) | 0.351 |
| | P for trend** | 0.235 | |
| PFUnDA | 1st (≤0.39) | 1.00 (reference) | |
| | 2nd ( > 0.39, 0.67) | 1.29 (0.58, 2.91) | 0.535 |
| | 3rd ( > 0.67, 1.09) | 0.96 (0.43, 2.16) | 0.929 |
| | 4th ( > 1.09) | 0.48 (0.20, 1.17) | 0.107 |
| | P for trend** | 0.159 | |
| PFDoDA | 1st (≤0.11) | 1.00 (reference) | |
| | 2nd ( > 0.11, 0.16) | 0.97 (0.45, 2.09) | 0.946 |
| | 3rd ( > 0.16, 0.22) | 0.64 (0.29, 1.41) | 0.264 |
| | 4th ( > 0.22) | 0.41 (0.18, 0.97) | 0.042 |
| | P for trend** | 0.147 | |
| PFHxS | 1st (≤0.87) | 1.00 (reference) | |
| | 2nd ( > 0.87, 1.41) | 2.83 (1.20, 6.69) | 0.018 |
| | 3rd ( > 1.41, 2.84) | 2.52 (1.04, 6.10) | 0.041 |
| | 4th ( > 2.84) | 2.14 (0.90, 5.09) | 0.084 |
| | P for trend** | 0.093 | |
| PFHpS | 1st (≤0.05) | 1.00 (reference) | |
| | 2nd ( > 0.05, 0.09) | 1.40 (0.64, 3.06) | 0.407 |
| | 3rd ( > 0.09, 0.14) | 1.44 (0.65, 3.19) | 0.364 |
| | 4th ( > 0.14) | 0.90 (0.39, 2.10) | 0.809 |
| | P for trend** | 0.590 | |
| PFOS | 1st (≤3.94) | 1.00 (reference) | |
| | 2nd ( > 3.94, 5.99) | 1.10 (0.49, 2.46) | 0.812 |
| | 3rd ( > 5.99, 9.11) | 1.10 (0.50, 2.41) | 0.819 |
| | 4th ( > 9.11) | 0.65 (0.28, 1.50) | 0.314 |

| | |
|---|---|
| P for trend** | 0.588 |

Abbreviations: OR, odds ratio; CI, confidence interval; PCOS, polycystic ovarian syndrome.

* Adjusted for age, employment status, educational level, active/passive smoking, pregnancy and delivery.

** P-value for test of trend across quartiles.
